# Letter from the Editor in Chief: 50 Years of Insights into Alcohol Research

**DOI:** 10.35946/arcr.v44.1.07

**Published:** 2024-12-16

**Authors:** Pamela J. Wernett

**Affiliations:** National Institute on Alcohol Abuse and Alcoholism, National Institutes of Health, Bethesda, Maryland

As we approach the close of 2024, a milestone year that marks the 50th anniversary of *Alcohol Research: Current Reviews (ARCR)*, I want to take a moment to reflect on the journal’s history and evolution with our readers. *ARCR* is the open-access, peer-reviewed publication of the National Institute on Alcohol Abuse and Alcoholism (NIAAA) at the National Institutes of Health. First published in 1974 by NIAAA as *Alcohol Health & Research World*, the journal’s name has since changed twice—to *Alcohol Research & Health* in 1999 and then to its current name in 2012.

I joined *ARCR* in 2018 and am honored to have witnessed the journal’s recent exponential growth firsthand. I have worked and continue to work alongside many dedicated individuals, including federal and contract staff, past and current members of the *ARCR* Editorial Advisory Board, authors, and peer reviewers. Over the past half century, so many individuals have contributed toward the journal’s ongoing goal of publishing high-quality articles that offer valuable insights into alcohol research.

**Figure:**
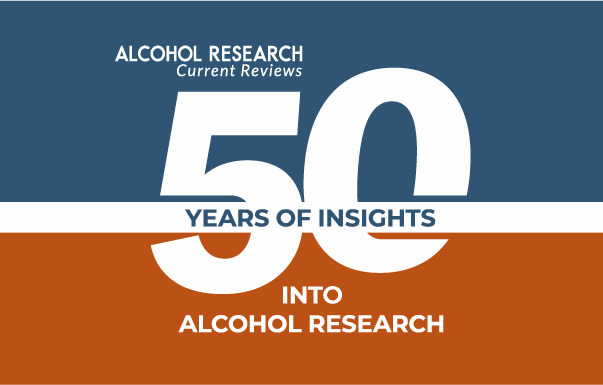

Looking back, it’s evident how far *ARCR* has come. The journal began by publishing quarterly print issues and started to go digital in 1994, providing articles online after publication of the print issue. This was the beginning of the journal’s open-access policy, which makes all articles freely accessible to the public. In 2020, *ARCR* became fully digital, ending print publication and adopting a continuous publication model. This pivotal change allows the journal to publish articles on a rolling basis, removing the delays associated with the previous “print first” model. Coupled with the journal’s open-access policy, it has greatly expanded *ARCR*’s reach by making articles more accessible to a larger number of readers around the world.

The evolution of the journal extends beyond its name changes, publication models, and level of accessibility. The earliest editions of the journal as *Alcohol Health & Research World* were primarily developed as a resource for health care and social service professionals interacting with individuals with alcohol use disorder and alcohol-related problems, as well as for lay audiences. As *Alcohol Research & Health*, the journal continued shifting its focus toward the research community with the inclusion of more peer-reviewed articles. In its current iteration, *ARCR* is fully peer-reviewed and geared toward a broad audience of scientists and clinicians, exclusively focused on publishing review articles. To further distinguish the journal as a reliable source of scientific information in the field of alcohol research, in 2021 *ARCR* revised the requirements for its narrative reviews, the journal’s nonexhaustive review type, to enhance transparency and rigor. In 2022, the *ARCR* editorial team introduced scoping reviews, the journal’s exhaustive review type that uses a standard format recognized in the field of peer-reviewed publication for increasing rigor and transparency.

Through its focus on increasing scientific rigor and accessibility, the journal has substantially enhanced its influence, as measured by its Journal Impact Factor^TM^ (JIF). *ARCR*’s JIF has risen over the years, especially in the last 5 years. In 2024, the journal received recognition again by being ranked first among all journals in the “substance abuse” category of the Social Sciences Citation Index,^1^ which represents a carefully selected and evaluated collection of journals that deliver the most influential scientific research information to users. This remarkable accomplishment is a testament to the journal’s increasing impact on the field of alcohol research.

Several new developments have been rolled out in 2024. During the COVID-19 pandemic, the *ARCR* editorial team recognized an opportunity to highlight quickly emerging fields of alcohol research. In response, the journal introduced a new article type called Perspective. There are two categories of Perspective articles. The first category includes concepts in alcohol research that present a forward-thinking view of an emergent area or a novel outlook on an established area of research, with the aim to guide and propel the field forward. The second category includes methodological considerations that discuss the latest developments, critiques, and best practices related to methodological and analytical tools relevant to alcohol research. *ARCR*’s first Perspective article is anticipated to be published in 2025. Additional developments launched this year include website features—“Curated Collections” and “News and Notes”—to make the *ARCR* website more user-friendly and informative. Even more exciting developments are underway for the journal. We encourage our readers to follow us on LinkedIn and to subscribe via our website to receive email notifications from *ARCR*.

**Figure:**
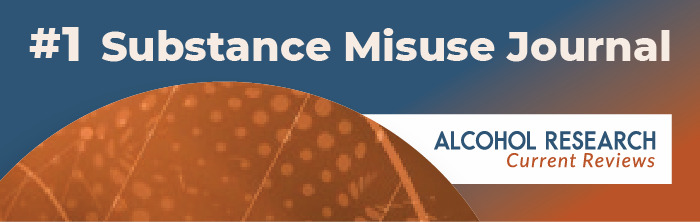

As we conclude our 50th anniversary celebrations, I would like to take this opportunity to thank our dedicated readers. *ARCR* remains committed to publishing articles that are accessible not only to alcohol researchers, but also to health care practitioners, educators, policymakers, and more. We do this for you.
